# Retinal Ischaemia in Diabetic Retinopathy: Understanding and Overcoming a Therapeutic Challenge

**DOI:** 10.3390/jcm12062406

**Published:** 2023-03-21

**Authors:** Ajay A. Mohite, Jennifer A. Perais, Philip McCullough, Noemi Lois

**Affiliations:** 1 Department of Ophthalmology, Belfast Health and Social Care Trust, Belfast BT12 6BA, UK; 2 Welcome-Wolfson Institute for Experimental Medicine, Queen’s University Belfast, Belfast BT9 7BL, UK

**Keywords:** diabetes, diabetic retinopathy, DR, retinal ischaemia, retinal nonperfusion, retinal capillary dropout, diabetic macular ischaemia, DMI, ultra-widefield fluorescein angiography, UWF FFA, FFA, optical coherence tomography angiography, OCTA, OCT angiography, OCT, retinal function

## Abstract

Background: Retinal ischaemia is present to a greater or lesser extent in all eyes with diabetic retinopathy (DR). Nonetheless, our understanding of its pathogenic mechanisms, risk factors, as well as other characteristics of retinal ischaemia in DR is very limited. To date, there is no treatment to revascularise ischaemic retina. Methods: Review of the literature highlighting the current knowledge on the topic of retinal ischaemia in DR, important observations made, and underlying gaps for which research is needed. Results: A very scarce number of clinical studies, mostly cross-sectional, have evaluated specifically retinal ischaemia in DR. Interindividual variability on its natural course and consequences, including the development of its major complications, namely diabetic macular ischaemia and proliferative diabetic retinopathy, have not been investigated. The in situ, surrounding, and distance effect of retinal ischaemia on retinal function and structure and its change over time remains also to be elucidated. Treatments to prevent the development of retinal ischaemia and, importantly, to achieve retinal reperfusion once capillary drop out has ensued, are very much needed and remain to be developed. Conclusion: Research into retinal ischaemia in diabetes should be a priority to save sight.

## 1. Introduction

Retinal ischaemia is a common and early event in diabetic retinopathy (DR) [1,2,3]. Thus, acellular capillaries are invariably seen early on in experimental models of DR and are likely also to be a very early manifestation in human DR [4,5]. Acellular capillaries are capillaries in which all that remains is the basement membrane following the loss of pericytes and endothelial cells [6,7,8]. Clinically, however, capillary drop-out may remain “silent” and undetected until it is perceivable with current diagnostic technologies, including fundus fluorescein angiography (FFA) or optical coherence tomography angiography (OCTA).

Despite the fact that the great majority of people with diabetes, if not all, will have retinal ischaemia to a greater or lesser degree, its extent is widely variable and the reasons for this remain unclear. Similarly, of all people with diabetes and DR, only a few will end up with the potentially visual threatening consequences of retinal ischaemia, namely ischaemic maculopathy (to which we will refer from now on as diabetic macular ischaemia, DMI) and proliferative diabetic retinopathy (PDR). Understanding why this may be the case will not only help identifying those at risk but also, and importantly, enhance our understanding of the pathogenic mechanisms underlying retinal ischaemia so that new treatments, including preventative therapies, are developed.

In this article, we compile and summarise knowledge on the pathophysiology of retinal ischaemia, its identification including pitfalls, risk factors, prevalence and progression, concordance between eyes, and its functional and structural implications and potential therapeutic strategies for it.

## 2. Pathogenesis of Retinal Capillary Drop-Out in Diabetes

Blood flow through the retina is controlled by a tightly interconnected and regulated neurovascular unit comprised of endothelial cells closely interlinked with pericytes in capillaries, vascular smooth muscle cells in arterioles, neurons (ganglion cells, bipolar, horizontal, and amacrine cells) and glial cells (Muller, microglia, and astrocytes) [9,10]. There is close talk and cell-to-cell interactions between endothelial cells and pericytes through gaps in the shared basement membrane [11]. These interactions, recently described as being of two types, direct contact points or pericyte-endothelial peg-and-socket formations [12], seem to be crucial to maintain a stable endothelium. Muller cells wrap around retinal capillaries; gaps in the basement membrane have been identified, however, which allow contact between Muller cells and pericytes [12], as well as areas lacking this Muller cell coverage, where neurons meet the vascular basement membrane [12]. Given the lack of autonomic innervation in the inner retina, the neurovascular unit and the “dialogue” among its different components are key to controlling blood flow, adjusting it depending on the requirements of the neuropile and maintaining it relatively constant despite changes in arterial and/or intraocular pressure [13,14].

**Figure 1:**
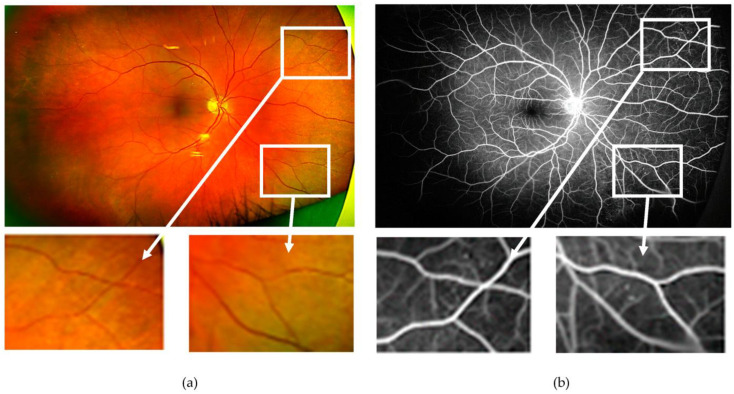
(**a**) Ultra-wide field pseudocolour image obtained from the right eye of a patient with diabetes with “no overt diabetic retinopathy”. Microaneurysms and small areas of capillary drop-out, however, are visible on the corresponding ultra-wide field fundus fluorescein angiogram (**b**). As an example, the two areas with no abnormalities are magnified as detected on the pseudocolour image (bottom left), which showed vascular changes on fluorescein angiography (bottom right).

Evidence suggests alterations in the normal regulatory mechanisms controlling blood flow occur early in the course of the disease in the diabetic retina, even before pathological structural abnormalities are seen (reviewed by Stitt et al. [7]). This is not surprising considering all components of the neurovascular unit are likely sensitive to the effects of hyperglycaemia [15,16]. A reduction in retinal blood flow, in the context of prolonged transit times through the retinal capillaries [17], has been documented in individuals with diabetes before the development of overt DR (defined as retinopathy that can be seen on fundus examination or fundus photographs) [17]. It should be noted, however, that many eyes labelled as having “no overt DR” (even in reading centres) have, nonetheless, vascular structural damage that could be revealed by FFA or OCTA imaging [18,19,20] (Figure 1). It has been observed that acute increases in blood glucose levels lead to parallel increases in retinal blood flow [17]; normalisation of glucose levels following the administration of insulin partially reversed the previously hyperglycaemic-increased blood flow through the retina [21]. As pathological changes progress in the diabetic retina, retinal blood flow seems to increase [22]. Circulatory changes in the diabetic retina, however, are complex and incompletely understood, despite the very large volume of research done over the years in this area (summarised by Schemetterer and Wolzt [23]).

A defective myogenic response (i.e., altered pressure autoregulation) may explain some of the early circulatory abnormalities in the diabetic retina. Thus, it was found in people with diabetes and no overt retinopathy that there was an unchanged or increased arteriolar diameter (instead of the normal expected decrease) following an incited increased in blood pressure [24]. A lack of compensatory vasoconstriction or even a paradoxical arteriolar vasodilation is followed by an increase in retinal capillary pressure and shear stress, which would be expected to cause subsequent endothelial cell damage and loss. Very recently published experimental work using vascular smooth muscle cells (VSMCs), including VSMCs from donors with diabetes, showed that the disruption of the myogenic vasoconstriction in retinal arterioles in diabetes was associated with a downregulation of transient receptor potential vanilloid subtype 2 (TRPV2) cation channels and loss of vascular smooth muscle cells stretch-activated cation currents [25]. Furthermore, heterozygous TRPV2 rats (*Trpv1^+/−^*), generated using CRISP-Cas9 editing technology, developed microvascular, glial, and neuronal lesions mimicking closely those observed in DR [25], including new vessel growth. The potential role of pericytes, which have also contractile properties modulating capillary blood flow, and how this may be affected by diabetes remains to be elucidated.

Besides alterations in retinal blood flow, other pathogenic mechanisms are known to contribute to early damage of retinal capillaries in diabetes. Thus, capillary occlusion by leukocytes was observed in early work undertaken by Schroder and colleagues [26]. Subsequent studies showed that this stasis of leukocytes (“leukostasis”) can lead to occlusion of retinal capillaries and reduced retinal perfusion (i.e., retinal ischaemia) [10,16,27,28]. Capillary drop-out with subsequent hypoxia, on its turn, would be expected to be followed by expression of a complex milieu of pro-inflammatory cytokines [29] and growth factors, such as hypoxia-inducible factor alpha (HIF-A) [30] and vascular endothelial growth factor (VEGF) [31] among many others. Under these circumstances, vascular leakage, diabetic macular oedema (DMO) and new vessel formation (PDR) may follow. The above may lead also to changes in the structure and composition of the vitreous or pre-retinal fluid in the presence of a complete or partial posterior vitreous detachment, additionally modulating the occurrence, progression, or regression of retinal ischaemia.

**Figure 2:**
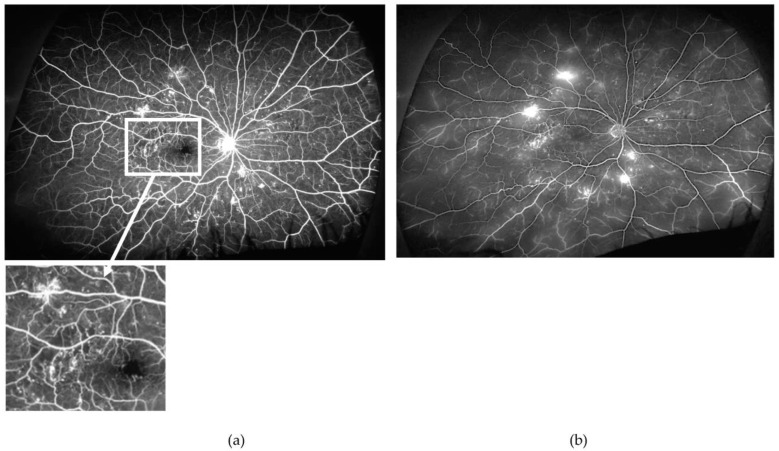
Arterio-venous (**a**) and late (**b**) phases of the ultra-wide field fundus fluorescein angiogram obtained from the right eye of a patient with proliferative diabetic retinopathy (PDR). Note the relatively well-perfused retina, except for very small areas of retinal ischaemia (example in inset) but several mid-peripheral new vessels elsewhere. Findings are very suggestive that other mechanisms, besides retinal ischaemia (e.g., inflammation), contribute to new vessel formation. Furthermore, findings highlight the great variability on the extension of areas of retinal ischaemia required for the occurrence of PDR.

**Figure 3:**
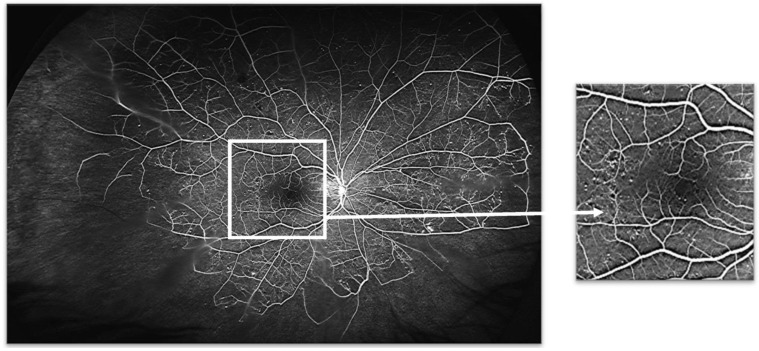
Venous phase of an ultra-wide field fundus fluorescein angiogram obtained from the right eye of a patient with non-proliferative diabetic retinopathy. Despite extensive midperipheral and peripheral retinal ischaemia, no new vessels are present. As findings show in Figure 2, this demonstrates also that there is great interindividual variability on the response of the retina to the insult of retinal ischaemia. Note the relatively well-preserved perifoveal capillaries (for details, see inset, right).

Interindividual variability in the above pathogenic events and compensatory mechanisms have not been investigated but should exist given the fact that only a small proportion of people with diabetes end up developing DMI, extensive retinal ischaemia, and PDR (Figure 2 and Figure 3).

## 3. Diagnostic Modalities for Detection of Diabetic Retinal Ischaemia

Two main diagnostic technologies allow visualisation of retinal blood vessels and are commonly used in clinical practice for the assessment of people with diabetes: ultrawide field fundus fluorescein angiography (UWF FFA) and OCTA. Other technologies could be used to measure retinal blood flow (e.g., colour Doppler and laser Doppler velocimetry). These, however, are not used clinically in the routine assessment of patients with diabetes and thus will not be reviewed herein.

### 3.1. Ultrawide Field Fundus Fluorescein Angiography (UWF FFA)

FFA is an excellent method for the evaluation of retinal ischaemia in DR. FFA requires the administration of a dye, fluorescein, in a peripheral vein. Images are then obtained as the fluorescein circulates throughout the retinal blood vessels, highlighting them. Areas of capillary nonperfusion can be recognised as dark areas lacking the dye.

FFA can be undertaken using standard fundus cameras. With these it is only possible to image a field of view of the retina of 30–50 degrees at a time with a single shot. If images are taken at the various gaze positions and with multiple shots, the four retinal quadrants can be visualised beyond the central retina. The Early Treatment Diabetic Retinopathy Study (ETDRS) proposed an imaging protocol for fundus images that includes seven overlapping fields [32]; this enable approximately 75 degrees of the retinal surface to be captured. With both eyes being affected in diabetes, this method becomes time consuming and challenging for the patient and technician acquiring the images as a minimum of 14 are required. Furthermore, given that the fluorescein circulates fast through the retinal blood vessels, it is not possible to document the exact moment in the circulatory phase in all regions as each subsequent image is captured at a slightly later stage than the previous one. For FFA studies, the ETDRS used only two central fields.

Most limitations of standard FFA have been overcome by the development of UWF FFA using the Optos scanning laser ophthalmoscope (Optos, PLC, Dunfermline, Scotland). This system produces a panoramic single image of up to 200 degrees, equating to ~82% of the retinal surface and approximately three times the area imaged using conventional 7-field ETDRS imaging [33,34,35]. With the newest instrument, a reduced amount of fluorescein is also required, with only 1.5 millilitres of 10% (100 mg/mL) fluorescein solution, maintaining excellent image quality. This is likely to reduce the risk even further of the already rare complications associated with FFA.

Once FFA is obtained, areas of retinal ischaemia are identified and measured. Traditionally, retinal ischaemia was measured in total number of disc areas (DA). With the advent of UWF imaging other methods have been proposed to quantify diabetic retinal ischaemia; however, to date there is no consensus regarding which one should be used. Most published studies evaluating retinal ischaemia in eyes of people with diabetes have used the ischaemic index. The ischaemic index is defined as a ratio of the area of capillary nonperfusion (capillary “drop-out”), identified in the arteriovenous phase of the FFA, to the total retinal area.

Areas of nonperfusion are most often delineated manually. Demarcating ischaemic areas manually to obtain an ischaemic index is time-consuming and potentially subjective. However, this technique seems to have excellent reproducibility with a reported inter-observer Lin correlation coefficient of 0.84 and a test-retest interclass correlation coefficient of 0.97 (95% CI 0.96, 0.98) [36]. Programmes and algorithms have also been devised to automatically identify areas of retinal ischaemia, albeit with limited success [37,38,39,40]. Indeed, detecting ischaemic areas is more challenging than detecting other features of DR, such as microaneurysms [39] and leakage [40]. This is due to variability in the appearance of ischaemic areas with regard to illumination, intensity, and uniformity [38] and has been found to be particularly problematic in the macula [40]. Buchanan and associates trained a machine learning algorithm to identify regions of retinal ischaemia in UWF FFAs based on a randomly selected dataset of 16 ground-truth image sequences with ischaemic areas having been manually demarcated by a retinal specialist (13 with haemorrhages, 9 with ischaemia, and 3 without pathology). Sequences consisted of 15–60 frames acquired up to 6 min from when fluorescein was injected. Ischaemic areas were considered present if fluorescence did not occur throughout the sequence. The algorithm correctly detected 70% of ischaemic regions [40]. Rasta and associates determined the sensitivity, specificity, and accuracy (not defined in the report) of automated detection of retinal ischaemia to be 81%, 78%, and 91%, respectively (ROC 0.796), based on standard FFA (the criteria for selecting the frame to be analysed was not reported) from 41 eyes of 80 patients with different levels of DR severity and compared them to the manual delineations of three clinical experts (no further information as provided in this regard), but poor quality images and those with “unusual appearances” or PDR without retinal ischaemia were excluded from the analyses [38]. A larger study by Son and associates evaluating 200 UWF FFAs (162 had non-proliferative DR (NPDR) and 38 had PDR) from 120 patients, reported a strong quantitative agreement between manually and automatically detected nonperfused areas (r = 0.863, *p* < 0.001) [39]. Poor quality images were excluded; the number of excluded images was not reported. Image distortion resulting from the stereographic projection of UWF images was not corrected for [39]. Automated detection of areas of retinal ischaemia often requires manual corrections [41].

Whether manually or automatically, the size of the area(s) of retinal ischaemia is determined in pixels or mm^2^ [36], and the ischaemic index can be then calculated. Although most studies have reported the value of the total ischaemic index for the eye where it is measured, it is also possible to determine an ischemic index for the various retinal areas (posterior pole, midperipheral, and far peripheral retina).

Nicholson and collaborators proposed a method using concentric rings of a progressively larger radius to determine size and location of capillary nonperfusion [37]. This method involves superimposing seven foveal-centred concentric rings, each divided into 12 segments, on UWF FFA images. Each segment is graded as “perfused”, “nonperfused”, or “ungradable” whenever more than 50% of the segment is categorised by either of these three categories; if each are the same in a segment, this segment is classified as “ungradable”. This method was shown to have as good reproducibility as the ischaemic index method [37].

**Figure 4:**
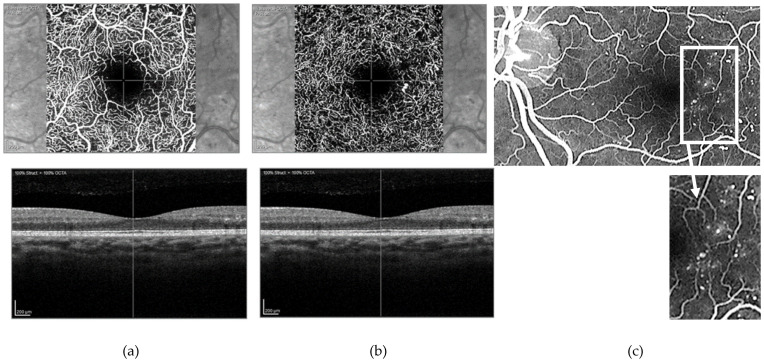
(**a**) Superficial and (**b**) deep retinal capillary plexuses as visualized using optical coherence tomography angiography (OCTA) and (**c**) corresponding fundus fluorescein angiogram (FFA). OCTA shows a well-preserved superficial capillary plexus (**a**) and what appears to be an area of capillary nonperfusion, supero-temporally to the foveal avascular zone (FAZ) and a microaneurysm temporally in the deep capillary plexus (**b**). FFA clearly shows multiple microaneurysms temporally to the FAZ ((**c**) and inset) not seen in the superficial capillary plexus on OCTA; one of these is imaged in the deep capillary plexus on OCTA imaging. This suggests that FFA, in contrast to what has been previously stated in the literature, is able to determine changes in the deep capillary plexus and, under certain circumstances at least (such as the case shown here), allows better visualization of retinal capillary changes than OCTA.

There are, however, general limitations of UWF FFA. Thus, although this diagnostic technology allows the assessment of blood flow through nearly the entire retinal vascular tree, it has been said to be able to image only the superficial capillary plexus [42,43] and, therefore, deep capillary plexus pathology may be missed. However, this may or may not be correct (Figure 4).

**Figure 5:**
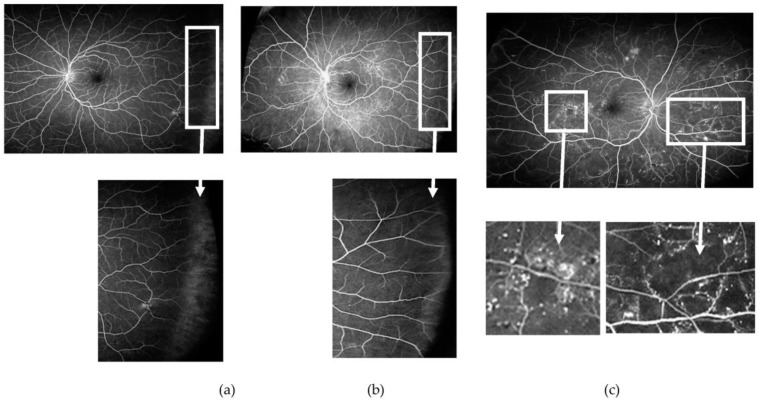
Venous phase of an ultrawide field fundus fluorescein angiogram obtained from three patients with non-proliferative diabetic retinopathy. Note in ((**a**) and corresponding inset) the lack of perfusion of the far peripheral temporal retina when compared with ((**b**) and corresponding inset) where blood vessels reached the far periphery. Interindividual differences with regard to the extension of the retinal vessels to the far peripheral retina may affect measures of retinal ischaemia if these areas of “nonperfusion” are considered “ischaemic”. In contrast, areas of ischaemia at other locations are usually well delineated and easier to identify, often surrounded by areas with multiple microaneurysms and microvascular changes ((**c**) and corresponding inset).

As the dye passes through the vascular system simultaneously through both eyes, it can be difficult to obtain good quality images from each eye of all circulatory phases. To ensure high-quality images with a full view of the retina are obtained, avoiding shadowing by the eyelashes, it is advisable that the eye being imaged is held widely open. This can be done by the operator obtaining the images, which may be difficult, or by an additional member of staff (requiring then two people to do the test), although the person holding the lids could be the same person that injects the dye. Depending on how late in the angiogram the images are taken, it may take 20–30 min to perform the test in full. A further issue is defining clearly what represents pathological retinal ischaemia, and this may be a limitation of many studies previously undertaken. When located at the posterior pole and midperipheral retina this may not be an issue as retinal ischaemia is usually clearly distinguished, flanked, and thus “flagged” by capillary abnormalities (Figure 3). However, determining whether peripheral “nonperfusion” is pathological (i.e., true retinal ischaemia) or not may be challenging (Figure 5). Nonetheless, UWF FFA currently seems to be the best method available for a detailed structural evaluation of the vasculature in eyes with DR and for the evaluation of areas of retinal ischaemia.

### 3.2. Optical Coherence Topography Angiography (OCTA)

OCTA allows for non-invasive imaging of the superficial and deep vascular plexuses of the retina (Figure 4a,b) [44,45]. It uses motion contrast technology to detect blood flow producing a snapshot of the retinal architecture with fine spatial resolution and enabling capture of capillary detail comparable to histological specimens [46]. This excellent spatial resolution has seen a growing uptake in OCTA for the evaluation of the diabetic eye. Coupled with high resolution OCT imaging, OCTA allows correlations between findings on retinal capillary plexuses with those in the neurosensory retina and retinal pigment epithelium (RPE) so that correlations between vasodegeneration and neurodegeneration in the diabetic retina can be better studied.

Analysis of OCTA images provides quantitative measures of vascular density of superficial and deep retinal capillary plexuses and enables the size and shape of the foveal avascular zone (FAZ) to be determined.

In healthy eyes, the FAZ has a well-demarcated round or oval appearance with a continuous margin in both the superficial and deep capillary plexuses. In DR, the FAZ is often pathologically enlarged due to the drop-out of surrounding capillaries [47] with loss of symmetry and circularity in both plexuses. Measurements of the FAZ area can provide an indication of diabetic microvascular changes. However, great variability exists in the size of the FAZ in the healthy state [48] and, therefore, it has been proposed that FAZ circularity may correlate more closely with capillary nonperfusion and macular ischaemia than the FAZ area [49].

The vessel density metric, widely used in published studies, is defined as the percentage of area occupied by vasculature in an en face image based on a binarised image and represents perfusion [50]. Vessel density measures are dependent on signal strength [51] and, therefore, require high-quality images but have been found to be reproducible in healthy eyes and eyes with DR. In a study by You and associates including 22 patients with retinal diseases and 15 healthy volunteers, intra-session (with each patient being asked to sit back between two scans while the OCT light beam was realigned) and inter-session (with an OCTA scan being repeated within two weeks of the first) reproducibility were determined using the Angiovue software metrics from 3 × 3 and 6 × 6 mm macular volume scans of the superficial capillary plexus. The intra-session reproducibility coefficient of variation for the different quadrants on 3 × 3 mm scans varied from 2.1 to 4.9% and 3.4 to 6.8% for healthy and diseased eyes, respectively, and for inter-session reproducibility 2.9 to 5.1% and 4.0 to 6.8%, respectively. The coefficients of variation were smaller in healthy compared to diseased eyes for intra-session compared to inter-session and for 3 × 3 compared to 6 × 6 mm scans [50]. An algorithm which detects inter-capillary spacing, however, appears to be more sensitive at identifying nonperfused areas [52] than those based on vessel density measurements.

Like UWF FFA, OCTA has also several limitations, the major one being its very limited field of view, which, at best, extends up to the vascular arcades and, therefore, is unable to capture pathological changes occurring in the mid-peripheral and peripheral retina. Given that these are the sites where retinal capillary drop-out is most commonly observed in diabetes [53,54,55,56], this represents a major weakness of this technology at present. Most currently used commercially available instruments have a field of view of 3 mm × 3 mm and 6 mm × 6 mm. Image resolution is the limiting factor in increasing the field of view because as it increases, resolution decreases due to lower sampling density. Montaging of these small areas imaged can be done, but the process is very time consuming with long scanning times that prove very difficult for patients. Furthermore, obtaining high-quality OCTA images is often challenging, especially if fixation is compromised. Other limitations of OCTA include motion and projection artefacts, which can make interpretation of images difficult and have been found to occur more frequently in eyes with pathology compared to healthy eyes [57]. OCTA has also been found to be inferior to FFA in detecting microaneurysms, and this is attributed to a reduced or turbulent blood flow in a microaneurysm, which fails to produce a decorrelation signal [58,59,60]. As technology advances, however, faster imaging capture and processing may facilitate easier access to a wider field of view and analysis at a high spatial resolution [61] improving OCTA imaging.

## 4. Risk Factors for Diabetic Retinal Ischaemia

Little knowledge exists regarding risk factors that may predispose people with diabetes to develop retinal ischaemia and, once this occurs, to increase the risk of its progression. In a retrospective cross-sectional study of 122 treatment naïve eyes of 70 patients with diabetes, Wessel and collaborators reported 62% of eyes had identifiable areas of retinal ischaemia as detected with UWF FFA [33]. In patients with identifiable diabetic retinal ischaemia, HbA1c was found to be statistically significantly associated with the proportion of retina found to be ischaemic (ß = 0.34, 95% CI 0.07 to 0.61, *p* = 0.01).

In another cross-sectional retrospective study including 651 treatment naïve eyes of 363 patients, male gender was positively associated with the total area of nonperfusion as detected with UWF FFA (difference, 15.72; 95%CI, 4.83–26.61; *p* = 0.005). Those with vitreous haemorrhage had increased total areas of nonperfusion (difference, 30.00; 95% CI, 5.26–54.75; *p* = 0.02). It was also reported that a threshold of total nonperfused area of 77.48 mm^2^ (95% confidence interval (CI), 54.24–92.66 mm^2^) was associated with the presence of PDR, but sensitivity and specificity were relatively low, specially the former (59.5% and 73.6%, respectively), and the CI was wide, suggesting that great variability exists among individuals [62].

A retrospective longitudinal study of 24 patients (43 eyes) with large areas of capillary nonperfusion, defined as more than 70% of the retinal area imaged, found that 75% of eyes had increased levels of creatinine and urea nitrogen and 37.5% of eyes had hypertension. Multivariable regression analysis, however, was not conducted to account for the influence of other potential risk factors [63].

Ra and associates measured VEGF levels in the aqueous humour of 47 patients with diabetes and treatment naïve PDR and found a statistically significant positive correlation between larger total, posterior, and peripheral areas of nonperfusion and VEGF levels (r = 0.575, 0.422, 0.558, respectively; all *p* ≤ 0.012) [64].

A cross-sectional, case-control study including 312 patients with type 2 diabetes, 159 with no overt DR, and 153 with DR identified low levels of haemoglobin (level not specified) (beta-coefficient = −0.49, *p* value = 0.001) to be associated with the presence of retinal ischaemia in multivariable regression analysis controlling for diabetes duration, sex, and erythrocyte level; however, ETDRS severity level was not controlled for [65].

## 5. Presence and Progression of Retinal Ischaemia in Diabetes

Early signs of retinal ischaemia are not visible clinically, on fundus examination, or standard fundus photography and, as a result, knowledge on the incidence, prevalence, and progression/regression of retinal ischaemia in the eyes of people with diabetes is very limited, as discussed below.

### 5.1. Presence of Retinal Ischaemia in Eyes of People with Diabetes

In the ETDRS, 3711 patients with mild to severe NPDR or early PDR had one eye randomised to early panretinal photocoagulation (PRP) and the other to observation until high-risk PDR developed, which allowed for the witnessing of the natural history of the disease in the initially untreated eye during the 1- to 5-year follow-up of the trial [66,67,68]. Analysis of FFAs (two horizontal 30-degree fields covering the macula (1F) and the nasal retina (2F)) at trial entry highlighted that the percentage of eyes with capillary loss increased with the DR severity score from mild NPDR (8%) to moderate (21–34%) and to severe (50%) but seemed to plateau in PDR from low-risk (48%) to high-risk (53%) PDR. As this was an interventional study, information on the prevalence of retinal ischaemia in the diabetic eye would be likely biased by case selection and, given that only two 30-degree central fields were used, would have been underestimated.

In a recently published prospective, multicentred, observational longitudinal study undertaken by the Diabetic Retinopathy Clinical Research Network (DRCR.net) (Protocol AA), including 508 eyes of people with type 1 or 2 diabetes andNPDR, UWF FFA was used to identify retinal nonperfusion [56]. At baseline, 91% of eyes were found to have this feature of DR. The fact that retinal nonperfusion is located predominantly in the mid-peripheral and peripheral retina may explain, at least partly, discrepancies in the prevalence of this feature in diabetic eyes between the above-mentioned studies [56].

In a retrospective cross-sectional study of 488 people with type 2 diabetes looking specifically at DMI, Sim and colleagues evaluated DMI in 408 eyes in which FFA macular-centred images were of adequate quality [69]. The ETDRS-DMI protocol was used to grade the severity of DMI. The authors found 39.7% of eyes had no DMI, 18.4% questionable, 25.2% mild, 11.0% moderate, and 5.6% had severe ischaemia. DMI was most prevalent in eyes with PDR (77.2%), followed by those with clinically significant macular oedema (CSMO) (69.4%), severe NPDR (59.7%), and DMO but without CSMO (55.8%) [69].

The above findings underline the great variability in the presence/absence, location, and extension of retinal ischaemia among individuals with diabetes.

### 5.2. Progression of Areas of Retinal Ischaemia in Eyes of People with Diabetes

There are a very limited number of prospective, longitudinal, and natural history studies evaluating specifically retinal ischaemia in DR as has been recently pointed out by Wykoff and associates [70]. Herein, we provide an account of those studies not presented in this recent review [70] and only point out important aspects of selected ones that were indeed included. Our interest is in specifically deciphering progression of ischaemia per se, rather than progression of DR on the ETDRS scale based on the degree of retinal ischaemia present, which was the main subject of most previous studies addressing retinal ischaemia in DR.

A small prospective case series (n = 20 eyes) evaluating longitudinally retinal nonperfusion with UWF FFA did not detect any increase in the retinal nonperfusion (RNP) index (total area of RNP divided by the total retinal area) over a one-year follow-up period. The DR status at baseline for these eyes was not provided [71]. Similarly, in the sham group of the RISE and RIDE, which served to evaluate the natural history of DMI over the 2-year course of the trial in people with DMO, FFA findings showed a non-statistically significant increase in macular nonperfusion over time (0.17 ± 0.43; 0.22 ± 0.63; and 0.27 ± 0.59-disc areas at baseline, months 12 and 24, respectively) [72]. It is unclear, however, whether changes of this size, even if not statistically significant, could be of clinical relevance, given the extraordinarily high metabolic demands of the macula. Nonetheless, these findings, if confirmed by other studies, are important as they would suggest that progression of RNP may be relatively slow in people with diabetes and, if so, if effective treatments were to become available there would likely be time enough to intervene and modify the course of the disease.

A prospective longitudinal cohort study including 30 eyes with NPDR (number of patients not reported) evaluated differences in rates of enlargement of areas of capillary nonperfusion as assessed using standard FFA depending on its retinal location (central, midperipheral, or peripheral) over a mean follow-up period of 11.6 months [53]. The rate of enlargement was determined as a percentage of the original value assessed by FFA, and it was found to be least rapid if peripheral (0.13% per month), followed by midperipheral (0.78% per month), central (4.78% per month), and generalised (the rate of enlargement in this group was not given; the authors explained this was due to the fact that progression in this group was such that treatment with PRP had to be given very soon after the initial examination) [53]. These findings could be potentially explained by the larger metabolic demands of the central retina populated also by a higher number of cells.

### 5.3. Reperfusion of Areas of Retinal Ischaemia

Few studies have reported on the occurrence of reperfusion of previously observed nonperfused areas. In a case report, Mohan and Kohner described and illustrated the occurrence of reperfusion in a previously extensive avascular area of the retina in a patient with PDR after extensive laser photocoagulation [73]. In a retrospective study including 94 eyes of 74 patients with DR followed at variable intervals for a mean period of two years using standard FFA but with panoramic and regional FFA images, Takahashi and associates documented reperfusion of occluded capillary beds in 69% of eyes [74]. This occurred by recanalization of previously occluded capillaries (in 34%) or by what they termed “intraretinal neovascularisation” (in 83%) in eyes that had not received laser photocoagulation. Systemic or local factors modulating the occurrence of retinal reperfusion were not investigated. The very high rates of spontaneous retinal reperfusion in this study are striking, and it seems surprising that this phenomenon has not been investigated further in detail in other well conducted studies, especially after the advent of UWF FFA. Understanding spontaneous retinal reperfusion would be key for the development of new therapies for retinal ischaemia and, very importantly, also for ischaemia in the brain.

A recently published case report documented spontaneous reperfusion of the paramacular area of a 35-year-old patient with diabetes within a seven-month period [75].

A review article on the effect of anti-VEGF injections on reperfusion in DR, which included 37 articles, concluded that most studies found no change in macular nonperfusion as assessed by FFA and OCTA, with the remainder finding enlargement of the FAZ post anti-VEGF treatment [76]. Improvement or stabilisation of nonperfusion was found in peripheral ischaemia (including the mid-periphery) in studies using UWF FFA. However, studies using wide field OCTA found no change in peripheral nonperfusion and no signs of reperfusion following anti-VEGF treatment. The review did acknowledge that studies were limited by their “retrospective design, small sample sizes, and short follow-up periods” [76].

## 6. Concordance of Retinal Ischaemia between Eyes

Given that diabetes is a systemic disease, it is reasonable to expect that DR (more specifically retinal ischaemia) would occur more or less symmetrically in both eyes. Genetic determinants, however, may play a role on its development, providing also potential susceptibility and symmetry between eyes. “Exposures” may also modulate its occurrence and progression.

There is little in the literature specifically addressing the question of concordance between eyes in the presence/absence, extension, location, and progression of retinal ischaemia in people with diabetes. Speilburg and colleagues retrospectively analysed UWF FFA images to assess interocular symmetry in 54 patients: one with mild, 9 with moderate and 22 with severe NPDR in both eyes; 16 with PDR in both eyes; 1 with moderate NPDR in one eye and PDR in the other; and 5 with severe NPDR in one eye and PDR in the other [77]. They obtained a single high-quality image of the arteriovenous phase from each eye, which was deemed to have the best clarity and field of view. This UWF FFA image was compared to the Optos UWF pseudo colour image to allow compensation for lash or eyelid artefacts. Paired images of both eyes were then graded by a single retina specialist for total gradable retinal area and peripheral nonperfusion area calculated by area of pixels using the V2 Vantage review software (Optos, PLC). This allowed them to calculate an ischaemic index (ISI) for each eye, by dividing area of nonperfused retina by total gradable retina and multiplying by 100; an ISI of 0 indicated absence of retinal ischaemia and an ISI of 100 indicated no retinal perfusion. They found no statistically significant difference (*p* = 0.85) in inter-eye ISI (mean ISI for the right and left eyes of 11.27 and 11.64, respectively). The mean absolute value (±SD) of inter-eye ISI difference was 4.46 ± 6.09; 72% and 93% of subjects had a difference in ISI of ≤5% and ≤10%, respectively, between eyes.

Niki and associates retrospectively analysed angiograms of 152 eyes from 111 patients who had NPDR [53]. Composite super-wide field FFAswere obtained, which involved acquiring several frames of different regions of the fundus. The prints were then enlarged and montaged to form a composite panoramic FFA of 130 degrees, 11 disc diameters (DD) temporal, and 8 DD nasal to the optic disc [55]. In 82 eyes, they had sufficient quality angiograms to analyse nonperfusion in each eye of each patient; nonperfusion was classified as peripheral, mid-peripheral, central, and generalised. Only four of 41 patients were noted to have an interocular discrepancy in the location of capillary nonperfusion. The authors concluded this difference may only portray a different stage of evolution of DR [53].

In the study by Sim and colleagues, two masked assessors graded images to determine the presence and severity of DMI as summarised above [69]; it is not clear, however, whether graders knew if eyes were paired at the time of assessment of FFAs. Of the 401 eyes with gradable images in both eyes, 249 (62%) had bilaterally symmetrical DMI as per the ETDRS-DMI grading. In those with asymmetric disease, the interocular discrepancy in ETDRS-DMI grading was ≤1 grade in 107 patients; 2 grades in 32 patients; 3 grades in 12 patients; and 4 grades in 1 patient [69].

Thus, although evidence is sparse, it suggests that retinal ischaemia in diabetes may affect similarly both eyes in most patients. This would be important if confirmed in well-designed studies as when testing new potential local therapies for retinal ischaemia, “fellow” eyes could be potentially used as controls of “treated” eyes.

## 7. Functional and Structural Implications of Diabetic Retinal Ischaemia

**Figure 6:**
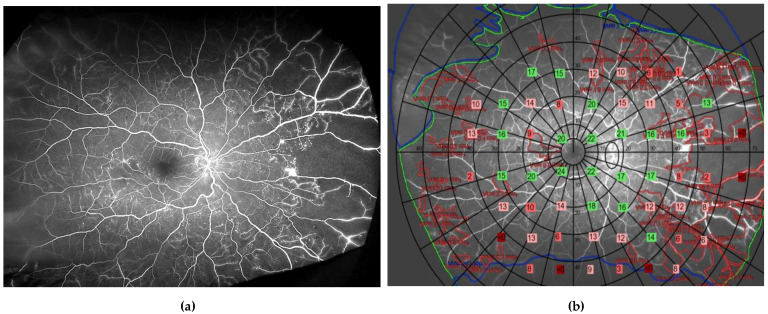
Ultrawide field fundus fluorescein angiogram (**a**) and same image with superimposed sensitivity values as derived from the Metrovision automated perimeter (57-point static test) (**b**). In green are depicted points with normal sensitivity; in light pink are those with deficits from 4 to 8 dB; in dark pink are deficits of >8 dB; and in red absolute deficits, when compared with those obtained in the corresponding locations in an age-matched group of healthy volunteers.

Several studies, reviewed recently by Wycoff and collaborators, evaluated the relationship between presence and extension of areas of retinal ischaemia and severity of DR [70]. The implications of retinal ischaemia on the functional and structural status of the affected retina, including point-to-point correlations, however, have been addressed in only a scarce number of studies. Multimodal imaging using UWF FFA, OCTA, OCT, and fundus autofluorescence coupled with functional testing (including measures of visual acuity and macular sensitivity) provided by macular microperimetry or more widely including also the midperipheral retina using newer technologies (Metrovision, https://metrovision.fr/contact-us.html, accessed on 28 December 2022) (Figure 6), as well as measures of metabolic activity (such as those given indirectly by retinal oximetry), are ideal to help us in understanding the relationship, including the temporal relationship between retinal ischaemia, function, and structure. These should be pursued.

Several early studies using Goldman or automated static perimeters and standard FFA showed reduced retinal sensitivity and scotomas in people with different stages of DR in areas of retinal ischaemia [78,79,80,81,82,83]. In some instances, however, retinal function remained preserved despite the presence of nonperfusion [78,80,83]. Bek noted that in certain cases where a blood vessel was crossing over an area of nonperfusion, retinal sensitivity adjacent to the blood vessel was maintained suggesting that diffusion of oxygen to the retina from this blood vessel was able to preserve retinal function [78]. Points with decreased retinal sensitivity were also found in perfused areas and it was proposed these may predict future capillary drop-out [79,81,83]. These studies were all cross-sectional and pointed out the need for prospective longitudinal studies to assess the relationship between retinal perfusion and function. Given that all were very early studies, no retinal structural data other than that gathered by fundus images were available.

Two studies by Unoki and colleagues [84] and Tsai and associates [85] investigated the relationship between retinal nonperfusion, function, and structure. Unoki et al. in a prospective cross-sectional study of 20 eyes (9 PDR and 11 severe NPDR) found reduced retinal sensitivity on microperimetry at sites of retinal ischaemia and in neighbouring areas. In 15 eyes imaged with OCT, thinning of the inner retina and deposition of hyperreflective material between the IS/OS and RPE were detected in areas of nonperfusion. In an early and very insightful meticulous histopathology study, Toke Bek [86] had previously described the loss of ganglion cells and thinning of the inner nuclear layer, as well as deposition of an eosinophilic material between photoreceptor outer segments and the RPE. Tsai et al. in a prospective longitudinal study evaluated the relationship between changes in macular perfusion using OCTA (3 × 3 mm scanning area), best corrected visual acuity (BCVA) (measured using Snellen charts), and mean retinal sensitivity using macular microperimetry (60 eyes of 31 participants with DR; 56 eyes available at the one-year follow-up evaluation). No point-to-point correlations, however, were sought. Masking of outcome assessors was not reported in either of these two studies.

Most studies seeking to establish relationships between function and retinal ischaemia included only visual acuity as a measure of retinal function. Thus, in their well conducted study (summarised above), Sim and colleagues found that although there were statistically significant differences in visual acuity between eyes with moderate and severe ETDRS-DMI grades when compared with those with lower grades, there was a large overlap among groups (median LogMAR of 0.2; IQR, 0–0.3; Snellen 20/32 in eyes with ‘‘none’’; 0.2 (IQR: 0–0.5; Snellen 20/32) in eyes with questionable; 0.2 (IQR: 0.2–0.5; Snellen 20/32); 0.5 (IQR: 0.2–0.6; Snellen 20/63) in eyes with moderate; and 0.6 (IQR: 0.3–0.8; Snellen 20/80) in eyes with severe ETDRS-DMI grades) [69]. No correlation was found between the FAZ area, measured in µm^2^, and visual acuity.

In a subsequent study, this group [87] found using a multivariable regression model, which included age, sex, eye, previous PRP, FAZ area, OCT-derived central retinal thickness, and indices of peripheral ischemia and leakage, that age (zero-order correlation coefficient [r] = 0.33, *p* = 0.03), FAZ area (r = 0.45, *p* = 0.02), and OCT-derived central retinal thickness measurements (r = 0.38, *p* = 0.01) (R^2^-adjusted = 0.36) were all associated with visual acuity.

Tsai and collaborators undertook a prospective cross-sectional study in patients with treated and stable PDR without centre-involving DMO, BCVA> 40 ETDRS letters, and OCTA evidence of DMI in at least one eye [88]. DMI was defined as either a foveal avascular zone (FAZ) measuring < 0.5 mm^2^ in the presence of ≥1 quadrant of parafoveal capillary drop-out or an enlarged/irregular FAZof ≥0.5 mm^2^ in the superficial vascular complex (SVC) [88]. A total of 125 eyes of 86 patients were assessed for structural changes in superficial and deep retinal vascular plexuses and their relationship with the presence of structural changes on OCT, including disorganisation of the retinal inner zones (DRIL) and ellipsoid zone (EZ) loss, as well as their implications on BCVA and low-luminance visual acuity. They found 83% of patients with DMI had good BCVA (≥70 ETDRS letters) and 48% good low luminance visual acuity (≥70 ETDRS letters). DRIL was more prevalent than EZ loss in their cohort (46% vs. 19%, respectively) and was found to have a more profound impact on visual acuity. DRIL was associated with a decrease in BCVA of 6.67 letters when compared to when neither DRIL nor EZ loss were present (95% CI, −10.55 to −4.82; *p* < 0.001). When there was concurrent DRIL and EZ loss, which was only found in 10% of eyes, the impact on vision was even more marked (decrease in BCVA of 13.22 letters; 95% CI, −18.85 to −7.59, *p* < 0.001) when compared to eyes with no structural abnormalities. Given the cross-sectional nature of the study, causal relationships between structural and functional changes cannot be established.

In a retrospective analysis (part of a larger study evaluating 78 treatment naïve patients with varying DR severity, with or without DMO), Antaki and associates [89] used UWF FFA images, OCTA, and OCT scans from 52 patients to evaluate functional (visual acuity with current refraction) and structural relationships related to diabetic retinal ischaemia. No correlation was found between eyes with predominantly central nonperfusion index and central subfield thickness on OCT in eyes with mild/moderate NPDR, although a moderate correlation was noted in those with severe NPDR (Spearman correlation coefficient (r_s_) = 0.496, *p* = 0.019). Central nonperfusion index was statistically significantly correlated with visual acuity (r_s_ 0.595, *p* = <0.001) in the severe NPDR group, but the association was no longer significant when controlling for central subfield thickness (*p* = 0.160). Eyes with PDR and predominantly central nonperfusion had a trend towards increased central subfield thickness (r_s_ = 0.412, *p* = 0.162) with no apparent association with visual acuity [89]. The peripheral nonperfusion index was not correlated to central subfield thickness for any severity of DR with the exception of PDR (r_s_ = −0.659, *p* = 0.014). Peripheral nonperfusion index and ratio were both correlated with visual acuity (r_s_ k = 0.549 and 0.626, *p* = 0.027 and 0.010, respectively). In this study, the “central” area was defined as an area including the optic nerve and the macular area extending up to the major vascular arcades, whereas the peripheral area was delineated from the edge of the central zone to the extent of visible retina (i.e., there was no distinction between midperipheral and peripheral retina). The nonperfusion index was estimated as the proportion of nonperfused to perfused retina, and the peripheral nonperfusion ratio was calculated by dividing the peripheral nonperfused area by the total central and peripheral area.

Sim and colleagues investigated the relationship between peripheral retinal ischaemia, FAZ area, and central retinal thickness in their study including 47 eyes of 47 patients with NPDR and PDR, with and without DMO [87]. When considering all eyes, the median FAZ area was statistically significantly larger in eyes with high peripheral ischemic index (0.56 mm^2^ (IQR = 0.86) when compared to those with low peripheral ischemic index (0.32 mm^2^ (IQR = 0.28)) (*p* = 0.02); differences, however, were no longer statistically significant when only PRP-treatment naïve eyes were included in the analysis (0.55 mm^2^ (IQR = 0.55) vs. 0.27 mm^2^ (IQR = 0.05); *p* = 0.06). A moderate correlation was found between peripheral ischemic index and FAZ area when considering all eyes (r = 0.49, *p* = 0.0001) or only those naïve to laser PRP (r = 0.55, *p* = 0.003). There were no statistically significant differences in the ETDRS-defined foveal central subfield thickness derived by OCT in eyes with a high or low peripheral ischemic index (226 µm (IQR = 94.5) compared to 267 µm (IQR = 75.0), respectively; *p* = 0.08). Eyes with a large FAZ had a statistically significantly thinner central retina compared to those with a small FAZ (median 217 µm; IQR = 81.8, compared to 272 µm, IQR = 36.0; *p* =0.02).

Nicholson and colleagues conducted a retrospective, post hoc, cross-sectional comparative analysis of images of patients enrolled in two completed randomised clinical trials in which retinal nonperfusion was evaluated using UWF FFA [90,91]. A single central UWF FFA image was used to investigate the threshold and location of retinal nonperfusion in eyes with NPDR, PDR, new vessels at the disc (NVD), and new vessels elsewhere (NVE). Using their developed method of concentric rings to measure areas of retinal ischaemia [37], they found that overall retinal nonperfusion was larger in eyes with PDR with a median retinal nonperfusion area of 147.9 DAs (95% CI, 127.4–173.5 DA) when compared to eyes with severe NPDR (median 67.8 DA; 95% CI, 44.2–107.3 DA) with a statistically significant difference in the median area of nonperfusion between NPDR and PDR (median 69.0 DA; 95% CI, 42.2–96.7 DA; *p* < 0.001). A nonperfusion threshold of >118.34 DA was found to have the highest specificity (84.8; 95% CI 68.1–94.9) combined with the highest sensitivity (66.1, 95% CI 52.6–77.9) to identify PDR. Confidence intervals were wide, suggesting great variability among individuals in the extension of retinal ischaemia required for the development of PDR. When evaluating eyes with NVD with or without NVE in comparison to NVE alone, eyes with NVD had a statistically significant increase in overall retinal ischaemia (191.8 DA (95% CI, 152.5–227.4 DA) compared to 127.4 DA (95% CI, 102.5–159.8 DA) (*p* < 0.001)). Eyes with NVD had a statistically significant increase in both median posterior pole (26.1 DA; 95% CI, 10.4–33.8 DA) and peripheral (157.0 DA; 95% CI, 143.2–193.5 DA) ischaemia when compared to eyes that had only NVE (median posterior pole ischaemia 10.4 DA, 95% CI, 1.8–13.9 DA; median peripheral ischaemia 113.0 DA, 95% CI, 94.3–140.3 DA). In this study, there was no distinction made between midperipheral and peripheral retinal ischaemia [92].

Shimizu and collaborators noted that capillary nonperfusion was more extensive in patients with iris neovascularisation when compared to those with disc and retinal neovascularisation [55]. Similarly, Bresnick and associates in a case series of eight patients with retinal ischaemia found that the development of rubeosis (8/15 eyes) was associated with more severe retinal ischaemia [93]. A more recent retrospective, cross-sectional study of 24 eyes (20 with PDR and 4 with central retinal vein occlusion) investigated the effect of patterns of capillary nonperfusion (posterior pole, mid peripheral, and far peripheral) in patients with recently diagnosed neovascular glaucoma. The ischaemic index, defined by dividing the proportion of nonperfusion by the total area of the zone, was found to be statistically significantly increased in the far periphery (93%) compared to the mid periphery (75%) and the posterior pole (35%) [94].

## 8. Current and Potential Future Strategies for Retinal Ischaemia

At present, there is no effective treatment to allow revascularization of the ischaemic retina. Many important aspects about this feature of DR, essential for the design of future trials testing new therapeutic interventions, remain to be elucidated. Thus, it is unclear what the speed of expansion of retinal ischaemia over time is, what are its main determinants, and what degree of interindividual variability should be expected. Similarly, it is currently unknown how quickly retinal ischaemia leads to a reduction in retinal function, not only regarding central vision, but also retinal sensitivity beyond visual acuity. If loss of function has occurred already as a result of capillary nonperfusion, when will it become irreversible? Can revascularisation be induced or capillary drop-out prevented by early intervention? These and several other questions require answers.

Once capillary drop-out has already occurred, restoring lost cells and preventing further cell demise would be required. Pericytes appear to be the earliest cell to drop-out followed by endothelial cell loss [7].

The field of vascular cell regeneration with therapeutic purposes has expanded exponentially over recent years. Mesenchymal stromal cells (MSCs) [95,96], less well-defined “CD34+” cells [97], myeloid angiogenic cells (MACs) [98] (also known as circulating angiogenic cells (CACs) or early endothelial progenitor cells (EPCs)), endothelial colony forming cells (ECFCs) [99,100] (also known as outgrowth endothelial cells (OECs)), and human induced pluripotent stem cell (iPSC)-derived endothelial cells (ECs) (iPSC-ECs) [101] have all been tested in experimental models of diabetes or retinal ischaemia, most frequently the oxygen-induced retinopathy (OIR) model and, in few instances, in early phase clinical trials in humans with the purpose of revascularizing the ischaemic retina (revised recently by Lechner et al. [102]). iPSC-derived pericytes [103] and adipose stell cells [104], which have characteristics of pericytes, including expression of some pericyte cell markers, have also been tested in limb ischaemia and OIR models, respectively.

Whereas some cells, including MSCs, CD34+ cells, and MACs, seem to exert their beneficial pro-angiogenic effects via a paracrine mechanism, ECFCs and iPSC-ECs, as well as iPSC-derived pericytes and pericyte-like adipose stem cells, seem to integrate in the microvasculature to truly “regenerate” it. Given that pericytes and endothelial cells are lost in a relatively parallel manner early on in diabetes, replacement of both is likely to be needed for therapeutic effects to be observed.

Clinical trials using any of the above cells, alone or in combination, would be complex to design. This complexity relates not only to the fact that diabetes is a systemic disease with multiple comorbidities to correct for, but also because of the different phenotypes and severity within DR, as well as the likely different progression rates of retinal ischaemia in individuals. Furthermore, current imaging modalities, although of high resolution, may not be sensitive enough to detect early potential beneficial effects of new therapies. As explained also in previous sections, the limited knowledge on the natural history of retinal ischaemia complicates the matter further.

## 9. Conclusions

The development and progression of diabetic retinal ischaemia is poorly understood. It is now widely accepted that abnormalities in all cellular components of the retinal neurovascular unit, together with altered inflammatory responses, all triggered by hyperglycaemia, leads to pericyte and endothelial cell loss and retinal ischaemia. Imaging methods to determine and measure areas of retinal ischaemia in the retina have improved in recent years; automated methods would help research progress faster in this area. A better understanding on risk factors determining retinal ischaemia, knowledge on its natural history, and how retinal ischaemia affects loss of function and alters its structure, including its time frame, would be of great benefit to inform and test the development of new therapeutics and should be pursued.

## 10. Search Strategy Used for Identification of Studies

We performed a literature search using PubMed and Google Scholar. The search strategy was developed around a combination of terminology pertaining to retinal ischaemia—‘diabetic retinal ischaemia (DRI)’, ‘diabetic macular ischaemia (DMI)’, ‘diabetic macular oedema (DMO)’, ‘retinal capillary non perfusion’, ‘retinal capillary drop-out’, and the following components: progression, reperfusion, risk factors, concordance of DR, ultra-wide field fundus fluorescein angiography, optical coherence tomography angiography (OCTA), retinal function, pathophysiology, pathogenesis of diabetic retinopathy, leukostasis, inflammation, multimodal imaging, microperimetry, anti-VEGF for DR, and vascular cell regeneration. Further articles were identified after review of references listed in pertinent studies included. Databases were screened with no time limitation (last search done 20 December 2022).

## Data Availability

Not applicable.
